# Gender differences in alcohol misuse and estimated blood alcohol levels among emergency department patients: implications for brief interventions

**DOI:** 10.1186/1940-0640-7-S1-A47

**Published:** 2012-10-09

**Authors:** Alexis Trillo, Roland Merchant, Janette Baird, Tao Liu, Ted Nirenberg

**Affiliations:** 1 Department of Emergency Medicine, Rhode Island Hospital, Alpert Medical School of Brown University, Providence, RI, USA; 2 Departments of Emergency Medicine and Community Health, Alpert Medical School of Brown University, Providence, RI, USA; 3 Brown University Center for Statistical Sciences, Providence, RI, USA; 4 Brown University Center for Alcohol and Addiction Studies, Brown University, Providence, RI, USA

## 

To ultimately create more effective brief interventions (BIs), we compared the extent of alcohol misuse severity and estimated blood alcohol levels (BALs) between male and female emergency department (ED) patients. We surveyed a random sample of nonintoxicated subcritically ill or injured 18-64 year-old English- or Spanish-speaking patients on randomly selected dates and times at two EDs in July-August 2009. Participants self-administered the Alcohol Use Disorders Identification Test (AUDIT) and a questionnaire about their alcohol use in the past 30 days. Using the formulae by Mathews and Miller, gender-specific BALs were estimated for participants according to their weight and the number of alcoholic drinks consumed on days when typically drinking and on days of heavy episodic drinking (≥5 drinks per occasion for men, ≥4 drinks per occasion for women). Gender-specific alcohol misuse severity levels (harmful, hazardous, or dependent) were calculated using AUDIT scores. Wilcoxon rank-sum and Pearson’s chi-squared tests were used to compare outcomes by gender. Of the 513 participants, 52% were women. Mean AUDIT scores were greater for men than women (7.5 versus 5.3; p < 0.001), although alcohol misuse severity levels were similar between men and women (24.4% versus 26.6% for hazardous drinking, 2.8% versus 2.2% for harmful drinking, and 6.5% versus 3.4% for dependence, respectively; p < 0.38). Men reported greater mean alcohol consumption than women when typically drinking (4.3 versus 3.3 drinks per day; p < 0.001) and during heavy episodic drinking (8.6 versus 5.3 drinks per occasion; p < 0.001). However, the mean BALs for men and women were similar when typically drinking (0.05 and 0.06, respectively; p < 0.13) and during heavy episodic drinking (0.13 and 0.12, respectively; p < 0.13). For future ED brief interventions, women may benefit from realizing that, despite drinking less alcohol on average than their male peers, they are reaching similar BALs with comparable levels of alcohol misuse.

